# Cyto-Histopathological Correlations in Pathology Diagnostics

**DOI:** 10.3390/diagnostics12071703

**Published:** 2022-07-13

**Authors:** Ivana Kholová

**Affiliations:** 1 Pathology, Fimlab Laboratories, Arvo Ylpön katu 4, 33520 Tampere, Finland; ivana.kholova@tuni.fi; 2 Pathology, Faculty of Medicine and Health Technology, Tampere University, Arvo Ylpön katu 34, 33520 Tampere, Finland

Cyto-histopathological correlation is a key player in measuring quality in a quality programme [1]. It was originally meant to measure cytology performance but can be valuable for biopsy practice, as well as laboratory process assessment [2].

The Special Issue collected together articles dealing with various aspects of cyto-histological correlation and its role in cytological diagnosis routine. 

Gynecological samples form the highest proportion of all cytological specimens. Its cyto-histological correlation as a part of quality assurance programmes was covered in the literature [1,2]. Asaturova et al. [3] applied cytology-histology correlation protocol guideline of the American Society of Cytopathology to 273 patients with cytology-histology agreement in 158 (57.9%) cases, minor discrepancies in 93 (34.1%) cases and major discrepancies in 21 (7.6%) cases. The reasons for discrepancies were colposcopy sampling errors, Pap test sampling errors, interpretative and screening errors. International standardization of cytology-histology correlation can help inter-laboratory comparisons and enhance self- and peer-learning [3]. Despite the trend to replace a conventional Pap test with HPV test, Rubeša-Mihaljević et al. [4] showed that diagnostic a Pap test on three slides was superior in the detection of high-grade squamous cell intraepithelial lesions in comparison to punch biopsy and endocervical curettage. 

Thyroid fine needle aspiration cytology is important and expanding field of cytology. Preoperative diagnosis of rare medullary carcinoma is essential for lesion management, so Asia-Pacific international multi-institutional cytomorphological analysis of smears prepared with different staining methods has impact on practice improvement and quality worldwide [5]. A Prof. Rossi led study [6] demonstrated another important issue that impact patient management and quality. In their series of 475 thyroid subcentimeter lesions, 57.2% suspected lesions were malignant and only a few samples were reported non-diagnostic. They summarized the importance of a multidisciplinary evaluation of subcentimeter thyroid lesions [6]. Comprehensive review on noninvasive follicular thyroid neoplasm with papillary-like nuclear features covers histopathological criteria, molecular profile, ultrasound features and cytological diagnosis [7]. Despite the entity not being possible to diagnose preoperatively, it influences risk of malignancy in several categories of the Bethesda System for Reporting Thyroid Cytopathology [7,8]. 

Pulmonary cytopathology is an emerging field with personalized medicine and immunotherapy related techniques application to cytology specimens in growing proportion. Swedish colleagues studied concordance of PD-L1 expression between cytology and histology specimens in pulmonary non-small cell carcinomas; the concordance was better in bronchoalveolar lavage and bronchial brushing than in pleural effusions [9]. This kind of study has essential output on patient management and quality of treatment. Another marker covered in the Special Issue is a new neuroendocrine biomarker insulinoma-associated protein 1 (INSM1) [10]. The nuclear expression of INSM1 is practical in all kinds of cytological preparations. The review by Maleki et al. concluded that INSM1 is a reliable sensitive and specific neuroendocrine marker in various localizations [10].

The contributory role of cell blocks (CBs) in salivary gland cytology classified according to The Milan System for reporting Salivary Gland Cytology (MSRSGC) was demonstrated by Tommola et al. [11]. The application of MSRSGC and CBs was found beneficial in a series of 365 fine needle aspiration specimens from a 2-year-period with an increase in diagnostic accuracy, and thus improving the patients´ treatment and management [11]. 

New cytopathology terminologies were also studied in three other papers [12,13,14]. The Sydney system for classification and reporting lymph node cytopathology was recently proposed by an expert panel [15]. Vigliar et al. [12] evaluated the Sydney system applicability in 300 fine needle aspirates of lymph nodes with 98.47% sensitivity, 95.33% specificity, and 97.06% accuracy. Additionally, the risk of malignancy stratification was performed.

The International System for Reporting Serous Fluid Cytology (TIS) was applied by Portuguese and Greek cytopathologists [13,14]. A series of 350 pleural effusions was retrospectively correlated with a corresponding pleural biopsy with 60.29% sensitivity and 98.56% specificity [13]. Pergaris et al. [14] calculated percentages and risk of malignancy in each TIS category separately for 528 pleural and 500 peritoneal effusion samples from a 3-year-period with recommendation of TIS utility in routine practice. 

Last, but not least I thank all authors, reviewers, editors and editorial office staff who contributed to the success of this Special Issue. 
